# CDC37L1 acts as a suppressor of migration and proliferation in gastric cancer by down-regulating CDK6

**DOI:** 10.7150/jca.56097

**Published:** 2021-03-31

**Authors:** Li Li, Xinyi Tao, Yandong Li, Yong Gao, Qinchuan Li

**Affiliations:** 1 Department of Oncology, Shanghai East Hospital, Tongji University School of Medicine, Shanghai 200120, China.; 2 Department of Medical Experimental Techniques, Jinzhou Medical University, Jinzhou 121001, China.; 3 Department of Thoracic Surgery, Shanghai East Hospital, Tongji University School of Medicine, Shanghai 200120, China.

**Keywords:** CDC37L1, Proliferation, Migration, Gastric cancer, CDK6.

## Abstract

The co-chaperone protein CDC37 (Cell division cycle 37) is well known to regulate multiple protein kinases and involved in tumor progression. However to date, little is known about its analogue CDC37L1 (Cell division cycle 37 like 1) in tumorigenesis. This study aimed to explore the expression and function of CDC37L1 in gastric cancer (GC). The immunohistochemical staining in a tissue microarray showed a weak expression of CDC37L1 in high grade GC tissues compared with low grade tissues. Consistently, data from online database analysis demonstrated that CDC37L1 level was decreased in stage 4 patients and low expression of CDC37L1 indicated a poor prognosis. Functional studies revealed that CDC37L1 could inhibit GC cell proliferation and migration in CCK8, EdU incorporation, colony formation and transwell assays. In the meantime, CDC37L1 also inhibited the tumorigenicity of GC cells in nude mice. Mechanistically, we found that CDC37L1 had an impact on CDK6 protein expression by western blotting. Palbociclib, a specific CDK4/6 inhibitor, was discovered to block the rapid growth phenotype of GC cells induced by CDC37L1 silencing. Taken together, these findings unveiled a tumor-suppressive role of CDC37L1 in GC, and CDK6 may act as a downstream effector in this process.

## Introduction

Gastric cancer (GC) remains the fourth most common malignant tumor and is one of the leading causes of cancer-related death worldwide 1, 2. From morphologic and molecular viewpoints, GC is a highly heterogeneous disease and is multifactorial in development 2, 3. Growing evidence have revealed that abnormalities in cell proliferation, differentiation and apoptosis are the main causes of tumor initiation and progression 4. The precise investigations in these processes may provide new understandings of GC development and therapeutic targets against this disease.

Previous studies have shown that CDC37 (Cell division cycle 37), as a molecular chaperone, interacts with heat shock protein 90 (HSP90) to regulate the folding and maturation of multiple protein kinases, most of which are oncogenes, such as cyclin dependent kinase 2 (CDK2), Raf-1 proto-oncogene, serine/threonine kinase (Raf-1) and cyclin dependent kinase 4 (CDK4) 5-9. CDC37 plays a major role in promoting the development of various cancers, including breast cancer, prostate cancer and liver cancer 6, 10, 11. CDC37 triggers breast cancer cell proliferation by regulating CDK2 activation and CDC37 depletion could reduce the activity of the mitogen-activated protein kinase 1 (Erk), AKT Serine/Threonine Kinase 1 (Akt), Mechanistic Target of Rapamycin Kinase (mTOR), resulting in prostate tumor cell growth suppression 12, 13. As well, CDC37 silencing also mediates the arrest of cell cycle progression in liver cancer 11. As an analogue of CDC37, CDC37L1 shares 31% homology with CDC37 protein 14. Reports have suggested that CDC37L1 expression is slightly decreased in HBV-related hepatocellular carcinoma tissues, specifically expressed in non-tumor nasopharyngeal epithelial tissues and down-expressed in nasopharyngeal carcinoma 15, 16. However, the role and mechanism of CDC37L1 in these tumors and other tumors remain to be explored.

In the present study, we used GC as a model to examine the expression and function of CDC37L1. Our immunohistochemical results, combined with the online analyses showed that CDC37L1 was lower at high grade GC samples, and the decreased expression of CDC37L1 was often accompanied by poor survival time. Furthermore, unlike CDC37, CDC37L1 had an inhibitory effect on the proliferation and migration of GC cells by CDK6 reduction.

## Materials and methods

### Materials

#### Cell lines

The human GC cell lines BGC-823, SGC-7901, MGC-803 and AGS were obtained from Cell Bank of Chinese Academy of Sciences, Shanghai, China.

#### Plasmids

The CDC37L1 cDNA (GenBank Accession Number: NM_017913.4) was cloned into pEX-3 to construct overexpression plasmid pEX-3-CDC37L1. The C-terminal of the expression product was fused 3×FLAG tag. The empty vector pEX-3 was used as a negative control. The overexpression plasmid (pEX-3-CDC37L1) was purchased from GenePharma (Shanghai, China).

#### siRNAs

The CDC37L1 siRNA was synthesis by GenePharma (Shanghai, China). The sense sequences are: siCDC37L1: 5'-AGCAGAGGAAGAAGGUUAU-3', and the negative control siNC: 5'-UUCUCCGAACGUGUCACGU-3'.

#### CDC37L1 overexpressed and silenced lentiviral particles

LV-CDC37L1 and LV-NC (negative control) for overexpression of CDC37L1; LV-shCDC37L1 and LV-shNC (negative control) for knockdown of CDC37L1; Two kinds of lentiviral particles were packaged and purchased from GenePharma (Shanghai, China). Stable cell lines were established by puromycin selection after lentivirus transduction.

#### Antibodies

Anti-CDC37L1 (16293-1-AP, Proteintech, China), anti-β-actin (sc-81178, Santa Cruz Biotechnology, Dallas, TX, USA), anti-CDK6 (14052-1-AP, Proteintech, China), anti-CDK4 (#12790, Cell Signaling Technology, USA), anti-Cyclin D1 (#2922, Cell Signaling Technology, USA), anti-FAK (66258-1-Ig, Proteintech, China), anti-P110 (#4255, Cell Signaling Technology, USA) and anti-mTOR (#2983, Cell Signaling Technology, USA).

#### Chemicals

Palbociclib (PD0332991) Isethionate was purchased from Selleck, Shanghai, China. MGC-803 and BGC-823 cells were treated with Palbociclib to perform cell counting kit-8 assay (5uM) and colony formation assay (1uM).

### Cell culture conditions and transfection

Cells were cultured in Dulbecco's modified Eagle's medium (DMEM; Corning, USA) supplemented with 10% FBS and 1% penicillin/streptomycin (Invitrogen, Carlsbad, CA, USA), and incubated at 37˚C in a 5% CO_2_ humidified incubator. Cell transfection was performed with lipofectamine 3000 (Invitrogen, USA) according to the manufacturer's instructions.

### Tissue microarray and immunohistochemical staining

Human GC tissue microarray was obtained from Outdo Biotech Company (Shanghai, China). The microarray (HStmA150CS02) contains 75 pairs of human GC specimens and the pathological information of corresponding patients. After immunohistochemical staining with CDC37L1 antibody (1:100), two pathologists independently evaluated the expression level of CDC37L1 according to the followed standard. The number of positive cells and staining intensity of each sample were evaluated, which were classified as weak, moderate or strong. The research and use of clinical samples were approved by the Medical Ethics Committees of Shanghai East Hospital, Tongji University.

### Western blot analysis

Cells were inoculated in 6-well plates and incubated for 24 hours. The protein was extracted with RIPA buffer. Before western blot analysis, protein lysates were diluted in 5×SDS loading buffer, and boiled for 5 minutes in metal bath. 10% sodium dodecyl sulphate polyacrylamide gel electrophoresis (SDS-PAGE) was used to electrophorese the protein extracts of cells. The protein on the gel was then transferred onto nitrocellulose filter (NC) membranes and blocked with 5% non-fat milk in PBST (0.05% Tween 20 in PBS) for 1 hour. The membrane and antibody were then incubated in primary antibodies overnight at 4°C, and washing for 15 minutes by PBST. Then incubated with secondary antibodies at room temperature for 1 hour and washing for 15 minutes by PBST. Detection of proteins was performed using the Odyssey Infrared Imaging System (Li-COR, USA).

### Cell proliferation assay

Cell counting kit-8 assay (CCK8) was performed to assess the ability of cell proliferation. GC cells were inoculated on 96-well plates and classified into control group and experimental group. After the cells adhered to the plate, 10 µl CCK8 (Dojindo Laboratories, Japan) was added to each well, and cultured at 37˚C for 75 minutes. Absorbance at 450 nm was measured in an automated plate reader, and the mean and standard deviation were calculated. The experiment was repeated three times under the same experimental conditions.

### Cell migration assay

Cell migration was investigated by Transwell chamber assay according to the instructions of the manufacturer. Briefly, the stable cells were trypsinized, and resuspended cells were mixed with serum-free DMEM and placed in upper chamber, and DMEM containing 10% FBS was added in the bottom chamber. The cells were incubated at 37°C for 24 hours. Then, the chambers were stained with crystal violet for 10 minutes and wiped with a cotton swab. The numbers of migratory cells in five random regions were counted and photographed under a microscope, and the experiment was repeated three times.

### Colony formation assay

Stable cells were seeded in 6-well plates at a density of 2×10³ per well, and cultured at 37°C for 2 weeks. The colonies were stained with crystal violet for 10 minutes, then photographed and counted, the experiment was repeated three times.

### Animal experiments

4-week-old BALB/c nude mice were inoculated with GC cells (BGC-823/LV-NC and BGC-823/LV-CDC37L1 or MGC-803/LV-NC and MGC-803/LV-CDC37L1) respectively. The mice were euthanized four weeks later, the tumors removed, photographed and weighed. Furthermore, the volume of tumors was monitored every week, and were calculated as follows: tumor volume (mm^3^) = (width^2^ × length)/2. All animals handling and experimental procedures were approved by the Ethics Committee of Shanghai East Hospital.

### EdU labelling and immunofluorescence

Stable GC cells were seeded in 12-well plates, cultured at 37°C for 24 hours. Then EdU labelling was operated following the manufacturer's procedures and stained with Apollo. After drying, the cells were photographed and counted with microscope. The experiments were repeated at least three times.

### Cell cycle assay

After exposing with or without Palbociclib for 24 h in 6-well plates, cells were collected and washed with precooled PBS. Subsequently, cells were fixed overnight with 70% ethanol precooled at -20℃. Fixed cells were washed at least three times, and resuspended with PBS containing RNase and propidium iodide (PI) solution for 15 min in the dark. Finally, cell cycle distribution of cells was detected by a flow cytometer (Beckman Coulter, CA), as well as was analyzed by *ModFit* LT software. Each experiment was repeated three times.

### Statistical analysis

Statistical data were analyzed by two-tailed Student's *t*-test or *χ*^2^ test using GraphPad-Prism (GraphPad Software, CA, USA). For all of the results, P<0.05 was considered as statistically significant.

## Results

### The expression of CDC37L1 is related to GC development by analyzing clinical samples

**Figure 1:**
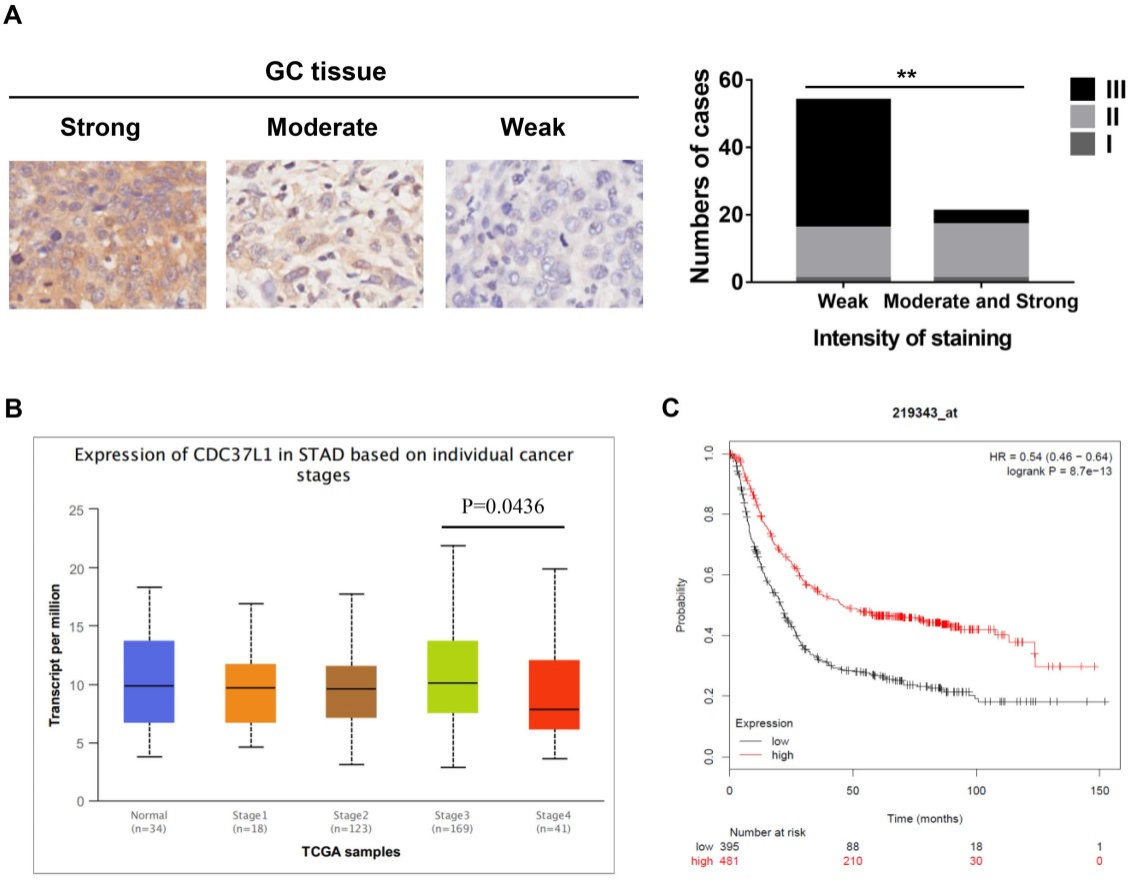
Expression of CDC37L1 in GC samples and relation with the clinicopathological factors. (a) Characterization of CDC37L1 protein expression in weak, moderate and strong GC specimens by immunohistochemistry staining (left). The correlation between staining of CDC37L1 and histological grade (right). (b) Differential transcriptional expression of CDC37L1 in normal samples and GC samples within individual cancer stages. (c) According to Kaplan-Meier survival curves, OS rate in patients with low expression of CDC37L1 (black line; n=395; P<0.01, log-rank test) was significantly lower than that in patients with high expression of CDC37L1 (red line; n=481).

To investigate the relationship between CDC37L1 expression and clinicopathological features, whose expression was examined through immunohistochemical approach in GC tissue microarray. The results implied that weaker expression of CDC37L1 is dramatically associated with higher histological grade (Figure 1A). Meanwhile, according to the UALCAN database analysis (http://ualcan.path.uab.edu), CDC37L1 expression was slightly related to cancer stages, suggesting the relatively low expression of CDC37L1 in stage 4 of GC (Figure 1B). Besides, Kaplan-Meier survival curves (https://kmplot.com/analysis/) showed that overall survival (OS) rate in patients with low expression of CDC37L1 was significantly poorer as compared to those with high expression of CDC37L1 (Figure 1C). The above data revealed that CDC37L1 might play a crucial role in GC progression.

### CDC37L1 inhibits GC cell proliferation in vitro

**Figure 2:**
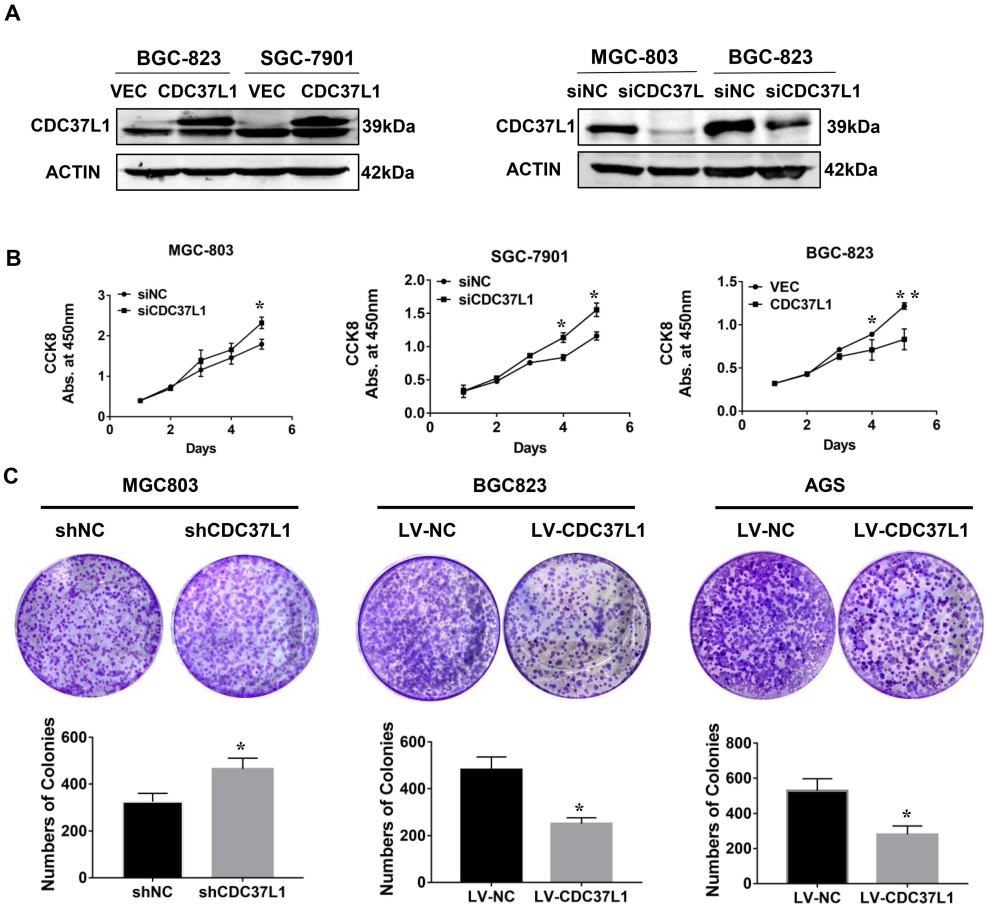
Effect of CDC37L1 expression on GC cell proliferation *in vitro*. (a) Western blot analysis results showed that CDC37L1 was effectively overexpressed or silenced in cells after transient transfecting GC cells with CDC37L1 plasmid or specific siRNAs. (b) Through CCK8 assays, knockdown of CDC37L1 promoted cell growth relative to control cells, and overexpression of CDC37L1 reduced cell proliferation. (c) Colony formation assays indicated that CDC37L1 inhibited the colony formation capacity of GC cells, and vice versa. Data are represented as mean ± SD of three independent experiments. *P<0.05, **P<0.01.

In order to explore the function of CDC37L1 in GC, we transiently transfected CDC37L1 plasmid or siRNA into GC cells for overexpression or knockdown experiments. Western blot results showed that CDC37L1 was successfully overexpressed or silenced in these cells (Figure 2A). Then, the effect of CDC37L1 on GC cell proliferation was determined through CCK8 assays. Knockdown of CDC37L1 markedly promoted cell growth relative to control cells, and cell growth rates in CDC37L1 overexpression group was strongly reduced compared with the control cells as indicated in Figure 2B. Moreover, to further examine the long-term effect of CDC37L1 on GC cell proliferation, we carried out colony formation assays. The results demonstrated that overexpression of CDC37L1 inhibited the colony formation capacity of GC cells (Figure 2C). Together, we found that CDC37L1 overexpression significantly inhibits GC cell proliferation *in vitro*.

### CDC37L1 suppresses the migration ability of GC cells

**Figure 3:**
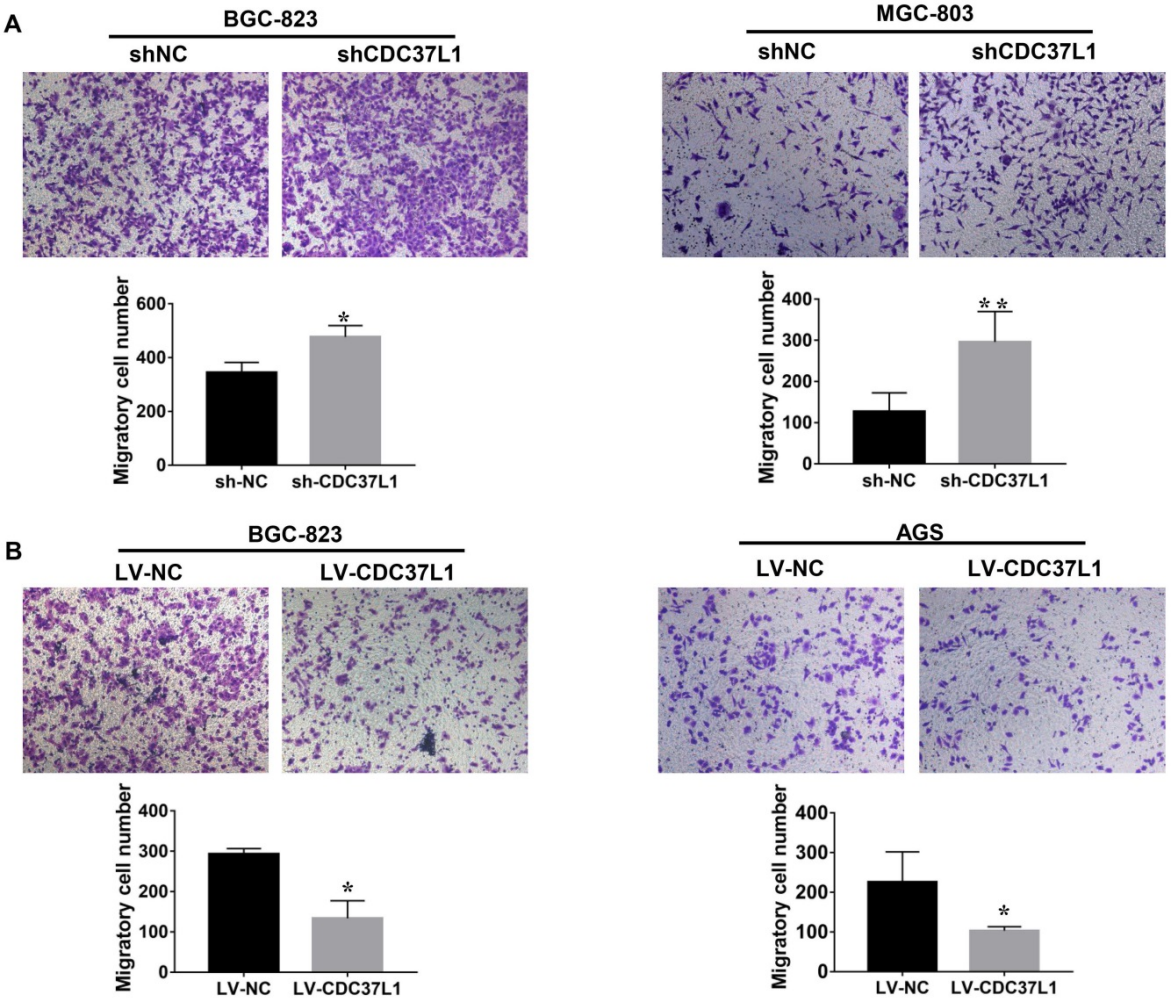
Expression of CDC37L1 suppresses the migration ability of GC cells. (a) The migrated cell numbers of BGC-823 and MGC-803 were significantly increased when CDC37L1 knockdown. (b) When CDC37L1 was overexpressed, the numbers of BGC-823 and AGS migrated cells were decreased compared with that in control cells. All data are expressed as mean ± SD. *P <0.05, **P < 0.01.

We also explored the effects of CDC37L1 on migration capacity of GC cells via Transwell chamber assays. As Figure 3A shown, knockdown of CDC37L1 significantly enhanced GC cell migration ability compared with control group. On the contrary, enforced expression of CDC37L1 resulted in reduced migration of GC cells relative to control group (Figure 3B). Collectively, our experiments elucidated that CDC37L1 could impair the migration of GC cells.

### CDC37L1 overexpression attenuates tumor growth in xenograft model

**Figure 4:**
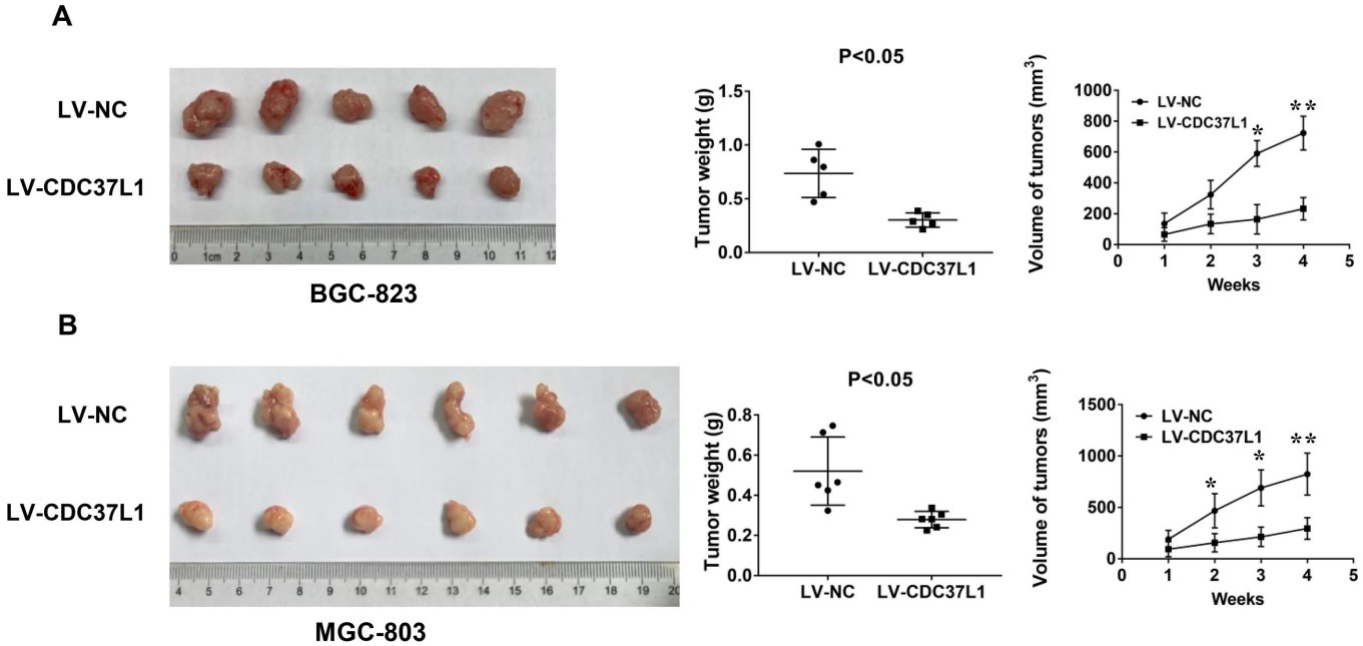
CDC37L1 inhibits tumor growth in xenograft model of GC. (a) Overexpression of CDC37L1 suppressed the tumorigenicity of BGC-823 cells by comparing the tumor size and weight. (b) CDC37L1 inhibited the tumorigenicity of MGC-803 cells. The data are represented as the mean ± standard deviation of three independent experiments. *P<0.05 and **P<0.01.

To further detect the effect of CDC37L1 expression on tumorigenicity of GC cells, we constructed a xenograft model. BGC-823 cells with or without CDC37L1 overexpression were subcutaneously injected into two separate groups of nude mice (n=5). Tumor volume in nude mice was measured weekly. After a month, the mice were sacrificed and tumors were weighed. The results showed that overexpression of CDC37L1 effectively suppressed the tumorigenicity of BGC-823 cells, reduced the tumor size and weight comparing with control cells (Figure 4A). Simultaneously, upregulating CDC37L1 or corresponding control MGC-803 cells were also inoculated into two separate groups of nude mice (n=6). The results were consistent with the above observations (Figure 4B). These consequences demonstrated that CDC37L1 inhibits tumor growth in xenograft model of GC.

### Knockdown of CDC37L1 increases CDK6 expression

**Figure 5:**
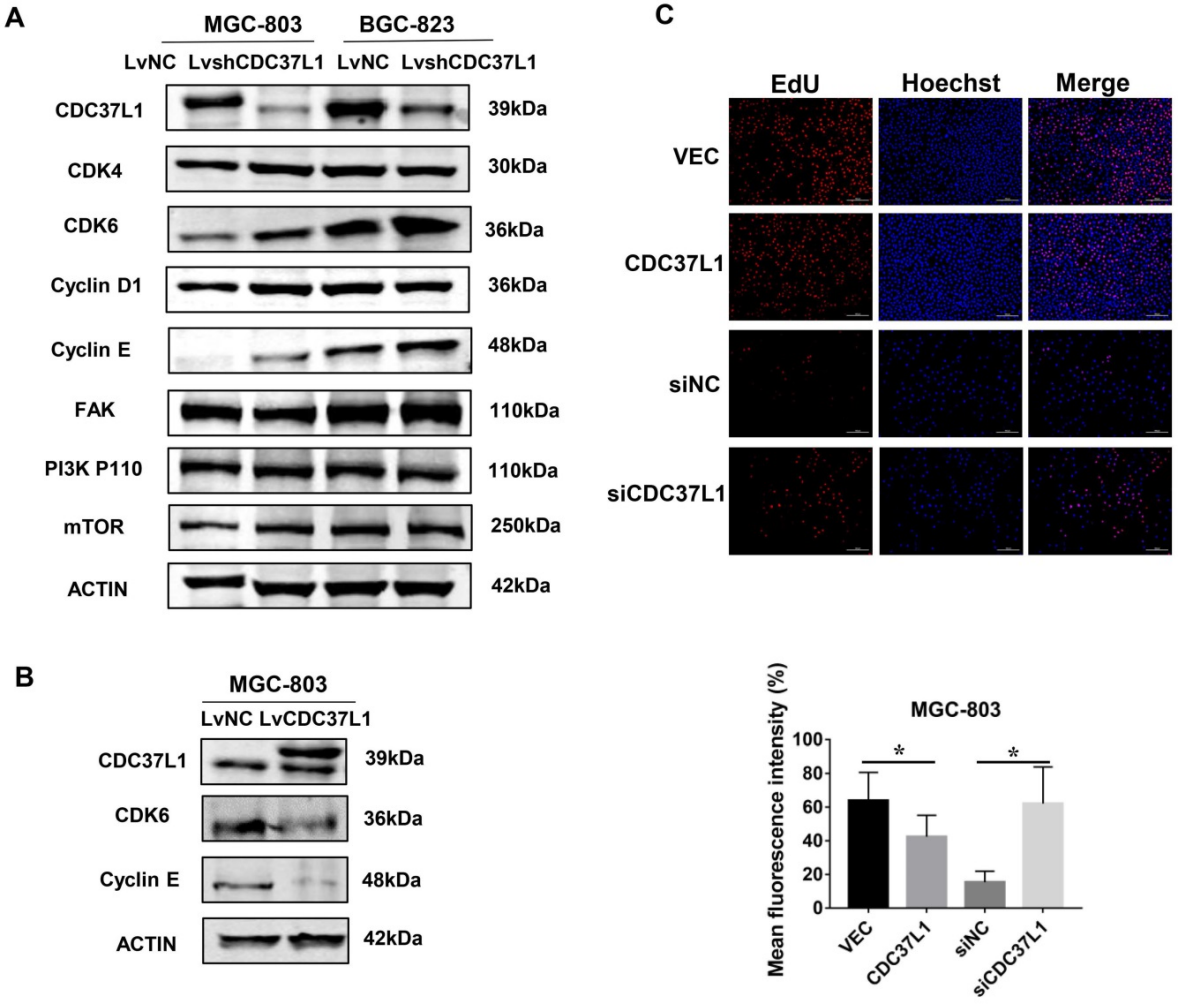
CDC37L1 suppresses CDK6 expression in GC cells. (a) Western blot assays showed that the protein level of CDK6 was up-regulated when CDC37L1 was knocked down in MGC-803 and BGC-823 cells. (b) CDK6 protein level was obviously reduced due to CDC37L1 overexpression in MGC-803 cells. (c) CDC37L1 inhibits GC cells growth by EdU incorporation assay, and vice versa. The histogram shows the mean and standard deviation of three independent experiments. *P<0.05 and **P<0.01.

In order to explore the molecular mechanism via which CDC37L1 inhibited GC cell proliferation and migration, we detected the expression of several cancer signaling pathway factors, such as CDK4, CDK6, Cyclin D1, FAK, PI3K-P110 and mTOR through western blot assays. As shown in Figure 5A, the protein levels of CDK6 in MGC-803 and BGC-823 cells were distinctly elevated after CDC37L1 blockade, while the others proteins remained unchanged. Subsequently, we reconfirmed the expression of CDK6 when CDC37L1 was overexpressed in MGC-803 cells. Western blot assay displayed that CDK6 protein levels were obviously down-regulated in overexpressing CDC37L1 cells (Figure 5B). CDK6, as a critical member of cyclin-dependent kinases, it has been well known that the role of CDK6 in promoting cancer development from previous studies. Therefore, our mechanistic studies illustrated that CDC37L1 attenuates the proliferation and migration of GC cells by inhibiting the expression of CDK6. Furthermore, we also employed EdU incorporation assays to detect the effect of CDC37L1 in cell growth. As the results shown that enforced expression of CDC37L1 in GC cells led to a lower percentage of EdU-positive cells than control groups, and silencing of CDC37L1 resulted in an increase EdU-positive cells (Figure 5C).

### Palbociclib hinders the anti-tumor role of CDC37L1

**Figure 6:**
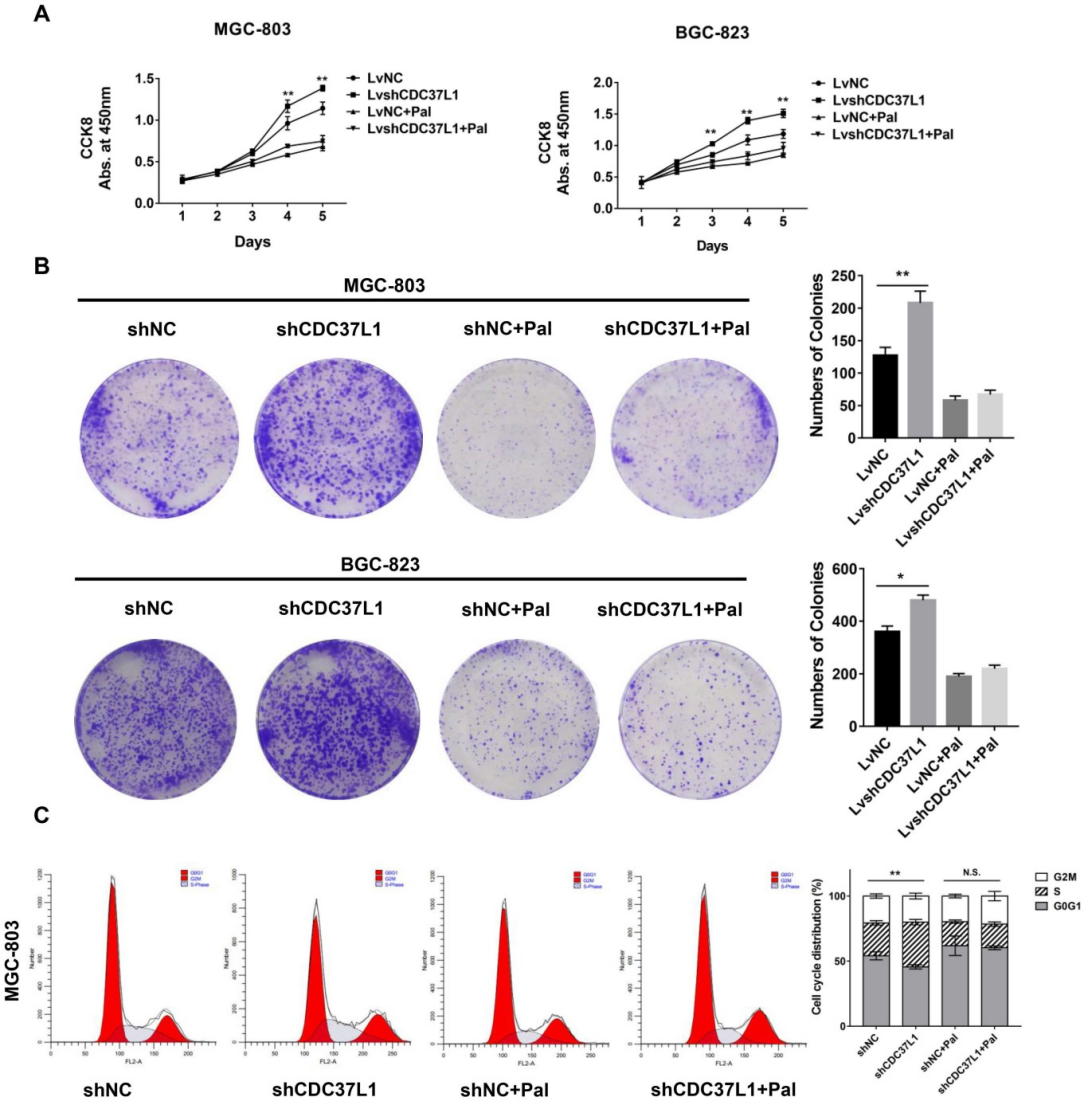
Palbociclib hinders the anti-tumor role of CDC37L1. (a)**-**(b) In stable silencing CDC37L1 or control group, MGC-803 and BGC-823 cells were treated with or without Palbociclib. CCK8 assay was conducted to examine cell proliferation. Colony formation assay was performed to test cell growth under such condition. Pictures of cell colony are presented (left panel), and the numbers of colonies were quantified (right panel). (c) Flow cytometry was used to detect the cell cycle distribution of CDC37L1 knockdown cells or control cells with and without Palbociclib treatment. All data are expressed as mean ± SD. *P <0.05, **P < 0.01.

To further confirm whether CDC37L1 suppresses cell growth through decreasing CDK6 expression in GC, we performed cell proliferation assays after GC cells were treated with Palbociclib, an inhibitor of CDK4/6. Strikingly, Palbociclib significantly abolished the effects of CDC37L1 on cell growth in MGC-803 and BGC-823 cells (Figure 6A). Similarly, CDC37L1 silencing increased the numbers of colonies compared with control, while there was no significant difference between the LvshCDC37L1 combined with Palbociclib group and the LvNC combined with Palbociclib group (Figure 6B). In addition, flow cytometry analysis revealed that CDC37L1 knockdown led to much more cells in S phase of cell cycle, whereas this increase in S phase cells could be hindered by Palbociclib treatment (Figure 6C). These results demonstrated that Palbociclib could inhibit CDC37L1 knockdown induced GC cell proliferation, further suggested the functional relevance between CDC37L1 and CDK6.

## Discussion

Abundant proofs have displayed that GC tumorigenesis was driven by the overexpression of major oncogenes or the down-regulation of anti-tumor genes. However, effective therapeutic targets are difficult to find due to characteristics of heterogeneity in GC 17. In our work, the role of CDC37L1 was uniquely studied in GC.

CDC37L1 (also known as HARC, CDC37B) is similar to CDC37 in protein structure, and contains 337 amino acids 18. On one hand, CDC37L1 and CDC37 respectively interact with HSP90 to enhance the binding of client proteins to HSP90 and assist protein folding 5, 19. On the other hand, CDC37L1 has been also found to heterodimerize with CDC37 *in vitro* and potentially contributed to the regulation of HSP90 function 18. In the field of oncology, it has been reported that CDC37L1 mRNA expression is lower in hepatocellular carcinoma tissues than in corresponding non-cancerous liver tissues, and higher expression of CDC37L1 is correlated with better outcomes of HCC patients 15, 20, 21. In addition to this, more functions of CDC37L1 in various kinds of cancers is waiting for exploiting. In the present work, the lower expression of CDC37L1 was observed in grade higher GC samples, as well as was accompanied by poor prognosis in patients. Moreover, functional assays demonstrated that CDC37L1 inhibits the ability of GC cells proliferation and migration. And* in vivo* results are consistent with the above. Collectively, these findings identified CDC37L1 as a tumor suppressor in the development of gastric cancer.

Cell cycle dysregulation is a common feature in human cancer 22. As a family of serine/threonine-specific protein kinases, cyclin and cyclin-dependent kinases (CDKs) have been studied maturely, which are characterized the key proteins to control cell cycle progression 23, 24. A lot of researches have indicated that CDKs must bind to cyclin subunit in order to be activated 24. CDKs consist of more than 20 members, among which CDK4 and CDK6 are the critical cell-cycle kinases. CDK6 plays crucial functions in the initiation of cell cycle and exerts significant actions on transcriptional regulation and differentiation 25-27. In general, activation of CDK6-Cyclin D complexes induces a transducer of cell-cycle progression, reinforces the transcription program to realize the regulation of gene expression and exerts an influence on tumor development 28, 29. Relevant studies have been published that CDK6 expression is up-regulated in pancreatic, prostate, bladder cancers, and so on 30. And CDK6 could be down-regulated by micro-RNAs, such as miR-129, miR-218, miR-449, to repress cell proliferation and migration in gastric cancer 31-33. In our study, western blot was used to search the downstream genes of CDC37L1 that affect the proliferation and migration of GC cells. We found that CDC37L1 leads to deregulation of CDK6 level in GC cells. Besides, the specific CDK4/6 inhibitor (Palbociclib) suppresses cell growth of GC cells caused by CDC37L1 deficiency.

In this study, we demonstrated for the first time that CDC37L1 plays an inhibiting role in proliferation and migration through down-regulating CDK6 expression in gastric cancer. Therefore, our data identify CDC37L1 as a potential drug target for the treatment of gastric cancer.

## Abbreviations

CDC37: Cell division cycle 37
CDC37L1: Cell division cycle 37 like 1
GC: gastric cancer
HSP90: Heat shock protein 90
CDK2: Cyclin dependent kinase 2
CDK4: Cyclin dependent kinase 4
Erk: Mitogen-activated protein kinase 1
Akt: AKT Serine/Threonine Kinase 1
mTOR: Mechanistic Target of Rapamycin Kinase
CCK8: Cell counting kit-8 assay
